# Time to Avoid Prophylactic Intra-abdominal Drainage in Acute Complicated Appendicitis: An Updated Systematic Review and Meta-Analysis

**DOI:** 10.7759/cureus.96500

**Published:** 2025-11-10

**Authors:** Mohamed Wael Ahmed, Yousra Elshoura, Neal Pun, Shafquat Zaman, Ahmed Alwetaidy, Cosmas Okeke, Georgios Kakaniaris, Pradeep Thomas, Najam Husain

**Affiliations:** 1 General Surgery, Queen's Hospital, University Hospitals of Derby and Burton NHS Foundation Trust, Staffordshire, GBR; 2 General Surgery, Kettering General Hospital, Kettering, GBR

**Keywords:** complicated acute appendicitis, duration of hospital stay, intra-abdominal collection, intra-abdominal drains, paralytic ileus, pediatric appendicitis, surgical drains

## Abstract

This systematic review and meta-analysis sought to compare clinical outcomes between patients who received abdominal drains and those who did not following appendicectomy for complicated appendicitis. A search across MEDLINE, PubMed, ScienceDirect, Embase, Scopus, and ClinicalTrials.gov identified 21 studies with 4,930 patients, including 1,809 with drains and 3,121 without. The meta-analysis revealed no significant statistical difference between the two groups regarding post-operative abdominal collection or mortality. The no-drain group yielded better results, with significantly fewer rates of surgical site infection, faecal fistulae, intestinal obstruction, and paralytic ileus, along with a shorter hospital stay. Additionally, a subgroup analysis of paediatric patients indicated an increased risk of abdominal collection in the drain group. Therefore, the evidence suggests that using an abdominal drain in appendectomy for complicated appendicitis increases post-operative complications, lengthens hospital stays, and elevates the risk of abdominal collection in children.

## Introduction and background

Acute appendicitis, characterised by inflammation of the vermiform appendix, is among the most common emergency surgical presentations worldwide [1,2]. Globally, it has an incidence of approximately 96.5 to 100 cases per 100,000 population, with a lifetime incidence of between 6.7% and 8.6% [1,3]. Although it can affect individuals at any age, it peaks between 10 and 30 years [1,2]. Making the diagnosis of acute appendicitis can be challenging, and typically involves a combination and synthesis of clinical assessment, laboratory, and radiological investigations [2].

Long considered a vestigial organ, the function of the appendix is currently being re-evaluated. The presence of lymphoid aggregates suggests a role in immune surveillance [4,5]. Moreover, it may serve as a reservoir for beneficial intestinal flora, replenishing the gut microbiota following disruptions through inflammatory bowel disorders, use of antibiotics, and gastrointestinal infections [4,5].

The lumen of the appendix can become obstructed by the presence of faecal material (fecalith), lymphoid hyperplasia, or malignancies. This rise in intraluminal pressure is believed to cause vascular congestion, stasis, bacterial overgrowth, and inflammation of the blind-ending appendix. In-time diagnosis and treatment are important to avoid the progression to necrosis and the perforation of the appendix [1].

Acute appendicitis can therefore be divided into simple/uncomplicated or complicated presentations, with complicated cases characterised by necrosis/gangrene, perforation, peritonitis, and abscess formation [2]. Differential diagnosis is broad, and the 'gold standard' treatment for acute appendicitis remains an emergency appendicectomy (performed through minimally invasive or open approaches) [1,2]. The benefits of laparoscopic surgery are well-established, including reduced pain, earlier mobilisation, shorter hospital stays, faster return to work, and improved overall quality of life scores [6]. Non-operative management in selected patients with uncomplicated appendicitis can be considered, but recurrence rates of ~39% within five years have been reported following conservative management [7].

Appendectomies performed for complicated appendicitis can be particularly challenging, and some surgeons may prefer to insert intra-abdominal drains prophylactically to reduce the risk of post-operative collections and abscess formation. However, the routine use of surgical drains remains controversial. They may be a source of significant post-operative pain/discomfort, limit mobility, and impair recovery. Additionally, drains may increase the risk of surgical site infections (SSIs), become blocked or difficult to remove, increase hospital stay, and rarely create faecal fistulas [8-11].

A number of studies have been published to investigate the impact of routine drain use following appendicectomies. A recent Cochrane review of randomised controlled trials (RCTs) and quasi-RCTs reported no evidence for clinical improvement with the use of abdominal drainage after appendicectomy for complicated appendicitis [8]. An earlier meta-analysis by Abu et al. (2022) showed no difference in the occurrence of intra-abdominal collections with the use of drains. Still, it did indicate an increased risk of SSIs, paralytic ileus, intestinal obstruction, and faecal fistulas [9]. Similarly, a meta-analysis focused on laparoscopic appendicectomy (Wu et al., 2024) and broader surgical evidence (Petrowsky et al., 2004) have also suggested no benefit from routine peritoneal drainage following appendicectomy for acute complicated cases; indeed, Petrowsky et al. argue for drain omission after appendicectomy irrespective of stage/grade of appendicitis [10,11].

The use of surgical drains remains a topic of debate. Our aim is to provide an up-to-date systematic review and meta-analysis to add to the existing evidence around the use of drains in patients undergoing appendicectomies for complicated appendicitis. In doing so, we hope to change current practice (promote evidence-based medicine), improve patient outcomes, and make better use of limited and finite healthcare resources.

## Review

Methods

The study and its methodology were planned and executed following the guidelines outlined in the Cochrane Handbook for Systematic Reviews of Interventions, in addition to the PRISMA (Preferred Reporting Items for Systematic Reviews and Meta-Analyses) recommendations for reporting [12,13]. The protocol is registered with the Prospective Register of Systematic Reviews (PROSPERO registration number: CRD420251140732) [14]. Two independent authors performed all stages of the review process, and a third author was consulted to resolve any discrepancies.

Paper selection was based on the PICO(S) (population, intervention, comparator, outcomes (study design)) format. We included patients of any age or gender with acute complicated appendicitis undergoing appendectomy (laparoscopic or open). The intervention was prophylactic intra-abdominal drain insertion, compared with no drain. The primary outcome was post-operative intra-abdominal collection/abscess formation, and secondary outcomes were SSI, paralytic ileus, bowel obstruction, length of hospital stay (LOS) in days, and mortality. The study design was a systematic review and meta-analysis of published comparative studies.

Literature Search Strategy

An electronic comprehensive database search was conducted using search operators and limits in PubMed, MEDLINE, ScienceDirect, Embase, Scopus, ClinicalTrials.gov, and the Cochrane Central Register of Controlled Trials (CENTRAL) up to and including 1 September 2025. The following search strategy was used: "drain" [All Fields] AND ("drainage" [MeSH Terms] OR "drainage" [All Fields] OR "drain" [All Fields] OR "lavage"[MeSH Terms] OR "lavage"[All Fields]) AND "laparoscopic appendicectomy" OR "laparoscopic appendectomy" [All Fields] OR "open appendicectomy" [All field] AND "acute appendicitis"[All Fields].

Eligibility and Study Selection

Two reviewers evaluated the titles and abstracts that were uncovered in the literature search independently. Afterwards, we obtained the full texts of pertinent articles and evaluated them based on our eligibility criteria. Any disagreements that arose during this process were settled through discussion and by consulting a third author.

All articles, including RCTs and comparative cohort studies (prospective/retrospective) satisfying our PICO requirements, were considered eligible for inclusion. We excluded conference abstracts, posters, case reports/series, letters to the editor, and studies that were single-arm and non-comparative.

Data Extraction and Collection

An electronic data extraction spreadsheet (Microsoft Excel, Microsoft Corporation, Redmond, WA) was created according to Cochrane's recommendations, pilot-tested, and adjusted accordingly [12]. The following information was extracted from each of the included studies: study-related data (author details, year of publication, location, study design, total number of patients, inclusion/exclusion criteria); baseline demographic and clinical information of the study population; and primary and secondary outcomes.

Assessment of Risk of Bias

We used the Cochrane Risk of Bias 2 (RoB2) tool to evaluate the included RCTs [15]. For observational studies, the methodological quality and risk of bias were assessed utilising the Newcastle-Ottawa Scale (NOS) [16]. For the former (Cochrane risk of bias), studies were judged as "high", "low", or "unclear" risk of bias across the following domains: randomisation, concealment of allocation, blinding of outcomes, blinding of participants and personnel, incomplete outcome data, selective reporting bias, and other sources of bias.

For observational data, a star-based scoring system (NOS) with a maximum score of nine points was used to assess the following: selection of study groups, comparability of these groups, ascertainment of the outcome of interest, and follow-up. Studies scoring nine points were judged to be at low risk of bias, those with scores of seven or eight were at medium risk, and those scoring six or less were at high risk, respectively.

Summary Measures and Data Synthesis

All statistical analyses were performed using RevMan 5.4 (Cochrane Collaboration, London, UK), and random-effects modelling was employed [17,18]. For dichotomous outcomes, the odds ratio (OR) was calculated using the Mantel-Haenszel method [19]. In instances where more than three included studies reported zero events in both arms for a given outcome, we used risk difference (RD) rather than OR. For continuous outcomes, we calculated mean differences (MDs) with 95% CIs. The well-practised equation by Hozo et al. [20] was used to estimate the mean and standard deviation (SD) in studies where continuous outcomes were reported as medians and interquartile ranges (IQRs). A p-value of <0.05 was considered statistically significant.

Heterogeneity between the studies was assessed using the Cochran Q test (χ2). We calculated the I^2^ statistic and interpreted it as follows: 0-50% might not be significant; 50-75% may represent moderate heterogeneity; and values between 75% and 100% represent substantial between-study heterogeneity [21].

To evaluate the robustness of our results, subgroup analysis was performed for studies that included laparoscopic appendicectomy (where outcomes were reported in three or more studies). In addition, sensitivity analysis was conducted through calculating the risk ratio (RR) or RD for dichotomous variables and 'leave-one-out' analysis to explore the influence of individual studies on pooled effect sizes and heterogeneity.

Results

Literature Search

**Figure 1:**
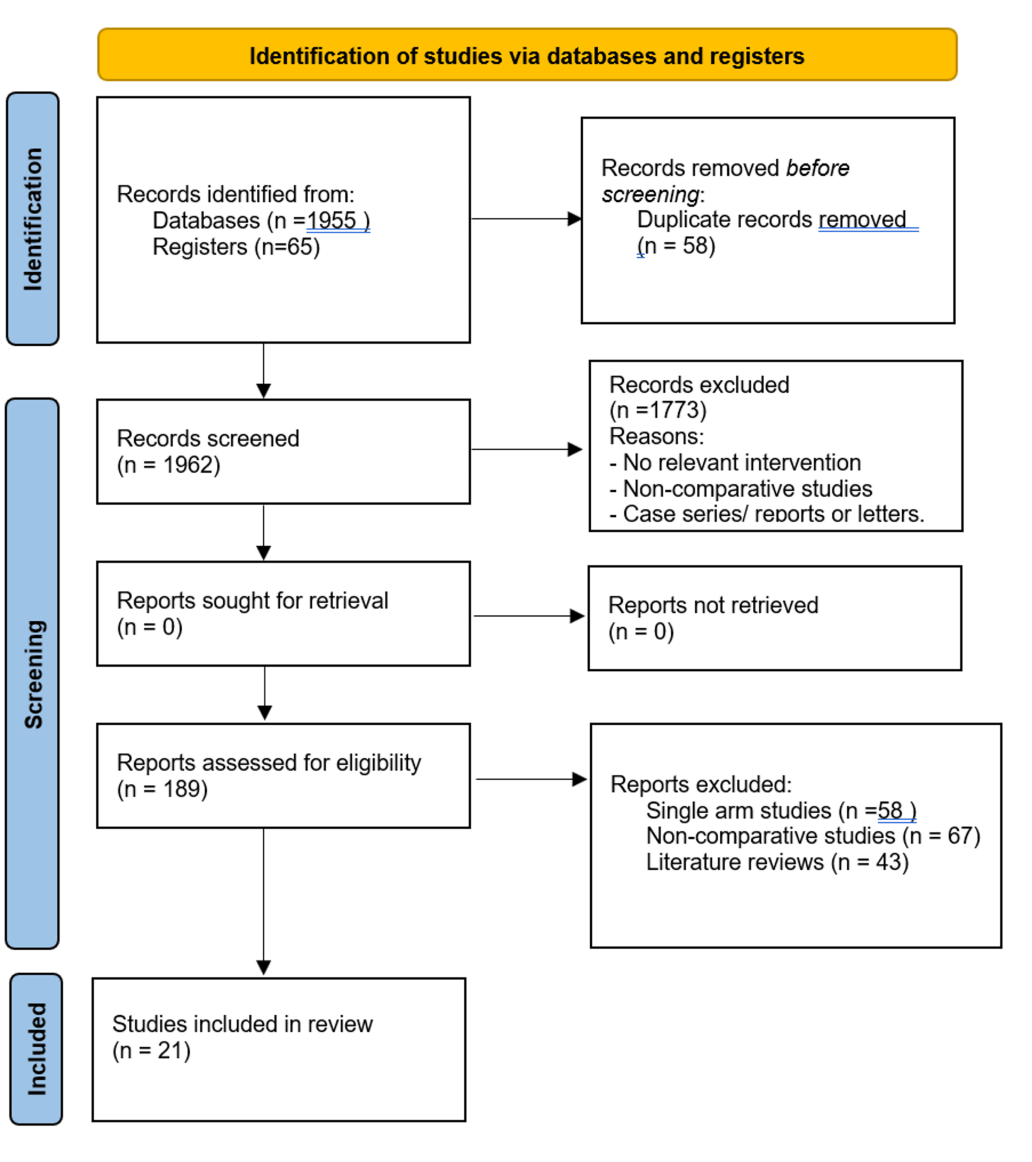
PRISMA flow chart. PRISMA: Preferred Reporting Items for Systematic Reviews and Meta-Analyses.

A total of 1,962 studies were identified following a comprehensive and systematic search of the above-mentioned electronic data sources. After reviewing titles/abstracts and excluding duplicates, 189 full-text articles remained, which were then screened and assessed against our inclusion criteria. Twenty-one studies meeting our eligibility criteria were included in the data synthesis, comprising a total of 4,930 patients divided into a drain (1,809 patients) and no-drain group (3,121 patients) following appendicectomy for acute complicated appendicitis [22-41]. The updated PRISMA flow chart is shown in Figure 1.

Study Characteristics

The studies included comprised a mix of RCTs [24-28,31,38] and observational data from a wide geographical area [22,23,29,30,32-37,39-41]. Both open and laparoscopic procedures were represented, and eight studies reported outcomes exclusively from a paediatric cohort [22,24,28,29,33,36,39,40]. For the remaining studies, three reported outcomes in an adult population [31,37,41], while the rest contained a mixture of adult/paediatric patients [23,25-27,30,32-36,38-40]. The baseline characteristics and the inclusion/exclusion criteria of the included studies are shown in Tables 1, 2.

**Table 1:** Baseline characteristics of the population. DG: drain group; NDG: no-drain group; WBC: white blood cells; NA: not available; SD: standard deviation; IQR: interquartile range.

Study	Age (years): Mean ± SD/Median (range/IQR)	Male-to-female ratio	Drain removal (days): Mean ± SD	WBC count: Mean ± SD/Median (range/IQR)	Antibiotics duration (days): Mean ± SD/Median (range/IQR)
Tsai et al., 2021 (JP drain group) [22]	DG: 9.3 ± 3.64	DG: 9:7	After the dressing had been infiltrated with a minimal amount of clear fluid	DG: 13.55 ± 6.44	NA - All patients received IV antibiotics until discharge
	NDG: 11.5 ± 3.59	NDG: 56:30		NDG: 17.62 ± 5.77	
				Tsai et al., 2021 (Penrose drain group) [22]		DG: 11.0 ± 3.92	DG: 13:6	Drain removed if clear fluid	DG: 17.28 ± 5.67	NA- All patients received IV antibiotics until discharge
NDG: 11.5 ± 3.59	NDG: 56:30	NDG: 17.62 ± 5.77								
Tran et al., 2023 [24]	DG: 9.01 ± 3.77	DG: 47:44	Drain removed ≤3 days (if <100 ml/24 h)	DG: 16.59 ± 4.86	DG: Median = 7.5 (IQR: 7–8; range: 6–9); NDG: Median = 6.5 (IQR: 6–7; range: 5–8)
	NDG: 9.2 ± 3.77	NDG: 60:33		NDG: 16.75 ± 4.55	
Liao et al., 2022 [23]	DG: 46.38 ± 19.01	DG: 125:67	NA- The criterion for drain removal was serosanguinous fluid	NA	NA
	NDG: 42.44 ± 19.93	NDG: 132:97			
Stone et al., 1978 [25]	All: 23.2 (0.3-82)	All: 159:124	5-10	NA	NA
Greenall et al., 1978 [26]	NA	DG: 31:17	NA	NA	NA
		NDG: 27:28			
Dandapat and Panda, 1992 [27]	NA	NA	NA	NA	NA
Tander et al., 2003 [28]	DG: 6.89 ± 3.5	DG: 50:20	1	NA	DG: 5
	NDG: 7.31 ± 3.4	NDG: 52:18			NDG: 5
Narci et al., 2007 [29]	DG: 8.7 ± 3.3	DG: 75:34			
	NDG: 8.5 ± 3.6	NDG: 75:42	NA	DG: 18.4 ± 5.8; NDG: 18.4 ± 7.0	DG: 9.5 ± 5.5; NDG: 7.7 ± 2.7
Allemann et al., 2011 [30]	DG: 38 (16-75)	DG: 72:58	2	DG: 14.0 (4-28.3)	NA
	NDG: 31 (16-71)	NDG: 83:67		NDG: 14.3 (4.1-23.6)	
Jani and Nyaga, 2011 [31]	DG: 49.99%	DG: 25:20	3	NA	DG: 5
	NDG: 50.01%	NDG: 21:24			NDG: 5
Pakula et al., 2014 [32]	DG: 32 ± 14	DG: 33:10	9 ± 5.4	DG: 15 ± 4.8	DG: 6.2 ± 4
	NDG: 29 ± 10	NDG: 25:80		NDG: 15.5 ± 5	NDG: 6.6 ± 4
Song and Jung, 2015 [33]	DG: 9.92± 4.25	DG: 55.6%:44.4%	NA	DG: 16.7 ± 5.96	DG: 6.38 ± 3.6
	NDG: 10.97± 4.04	NDG: 60.3%:39.7%		NDG: 15.7 ± 4.57	NDG: 3.87 ± 2.38
Schlottmann et al., 2016 [34]	DG: 43.3 (16-92)	DG: 36:20	NA	DG: 14.4 (6.3-23.4)	NA
	NDG: 43.1 (16-93)	NDG: 98:71		NDG: 15.3 (4.4-36.1)	
Abdulhamid and Sarker, 2018 [35]	DG: 31.75	DG: 47%-53%	NA	NA	NA
	NDG: 30.77	NDG: 53%:47%			
Aneiros Castro et al., 2018 [36]	DG: 7.57 ± 3.5	All: 63.1%:36.9%	NA	NA	DG: 7.51
	NDG: 8.07 ± 3.2				NDG: 6.61
Miranda-Rosales et al., 2019 [37]	DG: 35 (15-72)	DG: 60:40	NA	DG: 15.3 (10.0-19.0)	NA
	NDG: 36.76 (15-70)	NDG: 60:40		NDG: 15.6 (10.9-19.0)	
Fujishiro et al., 2021 [40]	NA	DG: 55.9:44.1	NA	NA	NA
		NDG: 59.9:40.1			
Nazarian et al., 2021 [41]	DG: 39.62 (17-82)	NA	NA	DG: 15.1	NA
	NDG: 37.42 (19-79)			NDG: 14.6	
Schmidt et al., 2020 [39]	DG: 10.34	DG: 18:14	NA	DG: 16.68	NA
	NDG: 10.70	NDG: 16:17		NDG: 16.62	
Mustafa et al., 2016 [38]	DG: 26.41 ± 6.24;	DG: 58.8%:41.1%	NA	NA	DG: 5
	NDG: 26.74 ± 4.97	NDG: 47.1%: 52.9%			NDG: 5

**Table 2:** Characteristics of included studies. DG: drain group; NDG: no-drain group; RCT: randomised controlled trial; NA: not available; IBD: inflammatory bowel disease; TUSPLA: trans-umbilical single-port laparoscopic appendectomy.

Study	Country	Study design	Patients (n)	Operation type	Inclusion/exclusion criteria and definition of complicated appendicitis
Tsai et al., 2021 (JP drain) [22]	Taiwan	Retrospective cohort (peds; TUSPLA)	Total: 121 (JP: 16, Penrose: 19, NDG: 86)	Open & laparoscopic	Inclusion: Patients undergoing appendicectomy for complicated appendicitis. Exclusion: Simple appendicitis. Definition: Perforated or gangrenous appendicitis with peritonitis
Tsai et al., 2021 (Penrose drain) [22]	Taiwan	Retrospective cohort (peds; TUSPLA)	(Subgroup of above, Penrose: 19)	Open & laparoscopic	Same as above, analysed separately to compare Penrose vs. JP drains
Liao et al., 2022 [23]	Taiwan	Retrospective cohort	Total: 421 (DG: 192, NDG: 229)	Laparoscopic	Inclusion: Patients with perforated appendicitis. Exclusion: Incidental appendectomy during other procedures, interval appendectomy. Definition: Intraoperative evidence of perforation
Tran et al., 2023 [24]	Vietnam	Prospective RCT (peds)	Total: 184 (DG: 91, NDG: 93)	Laparoscopic	Inclusion: Adults with perforated or gangrenous appendicitis. Exclusion: Patients with appendicular mass/abscess, interval appendectomy. Definition: Intraoperative perforation, gangrene, or generalised peritonitis
Stone et al., 1978 [25]	USA (Georgia)	Comparative	Total: 94 (DG: 49, NDG: 45)	Open	Inclusion/exclusion: NA. Definition: gangrenous or perforated appendicitis
Greenall et al., 1978 [26]	UK	RCT	Total: 103 (DG: 48, NDG: 55)	Open	Inclusion/exclusion: NA. Definition: NA
Dandapat and Panda, 1992 [27]	India	RCT	Total: 86 (DG: 40, NDG: 46)	Open	Inclusion/exclusion: NA. Definition: NA
Tander et al., 2003 [28]	Turkey	RCT (peds)	Total: 140 (DG: 70, NDG: 70)	Open	Inclusion: Paediatric cases with uncomplicated perforated appendicitis. Exclusion: appendicular mass/abscess. Definition: gross or microscopic perforation with no further peritoneal fluid discolouration after washout
Narci et al., 2007 [29]	Turkey	Retrospective (peds)	Total: 226 (DG: 109, NDG: 117)	Open	Inclusion: Children with macroscopic perforation. Exclusion: Appendix not visualised or drained without appendectomy. Definition: macroscopic perforation
Allemann et al., 2011 [30]	Switzerland	Case-matched study	Total: 260 (DG: 130, NDG: 130)	Laparoscopic	Exclusion: Uncomplicated acute appendicitis, generalised peritonitis, immunodeficiency, <16 years, incomplete data. Definition: Localised peritonitis, perforation, pus or fibrin, peri-appendicular abscess
Jani and Nyaga, 2011 [31]	Kenya	RCT	Total: 90 (DG: 45, NDG: 45)	Open	Inclusion: Advanced appendicular pathology, >13 years. Exclusion: Uncomplicated appendicitis, laparoscopic appendectomy. Definition: Perforated, mass or phlegmon
Pakula et al., 2014 [32]	USA	Retrospective cohort	Total: 148 (DG: 43, NDG: 105)	Laparoscopic	Inclusion: Gangrenous or perforated appendicitis per pathology/operative reports. Exclusion: Simple/suppurative appendicitis, interval appendectomy. Definition: Gangrenous and perforated appendicitis
Song and Jung, 2015 [33]	Korea	Retrospective cohort (peds)	Total: 342 (DG: 108, NDG: 234)	Open & laparoscopic	Inclusion: Children <18 years with acute appendicitis. Definition: Perforated appendicitis
Schlottmann et al., 2016 [34]	Argentina	Retrospective cohort	Total: 225 (DG: 56, NDG: 169)	Laparoscopic	Inclusion/exclusion: NA. Definition: Gangrenous/perforated appendicitis with peritonitis
Abdulhamid and Sarker, 2018 [35]	Iraq	Retrospective cohort	Total: 227 (DG: 114, NDG: 113)	Open	Inclusion: Open appendectomy for complicated appendicitis, all ages. Definition: perforated with localised abscess formation
Aneiros Castro et al., 2018 [36]	Spain	Retrospective cohort (peds)	Total: 142 (DG: 79, NDG: 63)	Laparoscopic	Inclusion: Laparoscopic appendectomy, excluded interval appendectomy. Definition: macroscopic hole in the appendix at surgery
Miranda-Rosales et al., 2019 [37]	Peru	Retrospective cohort	Total: 150 (DG: 50, NDG: 100)	Open	Inclusion: >18 years with complicated appendicitis. Exclusion: Laparoscopic appendectomy, anticoagulation, immunocompromised, pregnancy. Definition: Peritonitis or appendicular abscess
Mustafa et al., 2016 [38]	Pakistan	RCT	Total: 68 (DG: 34, NDG: 34)	Open	Exclusion: Immunocompromised, generalised peritonitis. Definition: Intra-operative perforated appendicitis
Schmidt et al., 2020 [39]	Germany	Retrospective cohort (peds)	Total: 65 (DG: 32, NDG: 33)	Open: 11; laparoscopic: 55	Inclusion: Age 2–17 years with perforated appendicitis. Exclusion: Severe neuro dysfunction, IBD. Definition: Perforated appendicitis on histology
Fujishiro et al., 2021 [40]	Japan	Propensity-matched (peds) study	Total: 1,762 (DG: 485, NDG: 1304)	Open: 346; laparoscopic: 958	Inclusion: Children ≤15 years with complicated appendicitis. Exclusion: Interval appendectomy. Definition: Perforation, gangrene, abscess
Nazarian et al., 2021 [41]	UK	Retrospective cohort	Total: 76 (DG: 26, NDG: 50)	Laparoscopic	Inclusion: ≥16 years with complicated appendicitis. Exclusion: Caecal/appendicular malignancy. Definition: Histologically gangrenous/perforated appendicitis

Assessment of Methodology and Risk of Bias

The methodological quality assessment of our observational studies is highlighted in Tables 3, 4. Two studies were assessed and found to have a low risk of bias [35,40], and the remainder scored as moderate risk.

**Table 3:** Methodological quality assessment of the observational studies via the Newcastle Ottawa Scale. Symbol (*) is the number of points given to each study according to the Newcastle-Ottawa scale (* gives one point, and ** gives two points).

Study	Is the case definition adequate?	Representativeness of the cases	Selection of controls	Definition of controls	Comparability of cases and controls on the basis of the design or analysis	Ascertainment of exposure	Same method of ascertainment for cases and controls	Non-response rate	Total
Stone et al., 1978 [25]	*	*		*	*	*	*	*	7
Narci et al., 2007 [29]	*	*		*	**	*	*	*	8
Allemann et al., 2011 [30]	*	*		*	**	*	*	*	8
Pakula et al., 2014 [32]	*	*		*	**	*	*	*	8
Song and Jung, 2015 [33]	*	*		*	*	*	*	*	7
Schlottmann et al., 2016 [34]	*	*		*	**	*	*	*	8
Abdulhamid and Sarker, 2018 [35]	*	*	*	*	**	*	*	*	9
Aneiros Castro et al., 2018 [36]	*	*		*	**	*	*	*	8
Miranda-Rosales et al., 2019 [37]	*	*		*	**	*	*	*	8
Fujishiro et al., 2021 [40]	*	*	*	*	**	*	*	*	9
Nazarian et al., 2021 [41]	*	*		*	**	*	*	*	8
Schmidt et al., 2020 [39]	*	*		*	**	*	*	*	8

**Table 4:** Methodological quality of the cohort studies assessed with the Newcastle Ottawa Scale. Symbol (*) is the number of points given to each study according to the Newcastle-Ottawa scale (* gives one point, and ** gives two points).

Study	Representativeness of the exposed cohort	Selection of the non-exposed cohort	Ascertainment of exposure	Outcome not present at start	Comparability of cohorts	Assessment of outcome	Follow-up long enough	Adequacy of follow-up	Total
Tsai et al., 2021 [22]	*	*	*	*	*	*	*	*	8
Liao et al., 2022 [23]	*	*	*	*	**	*	*	*	9

**Figure 2:**
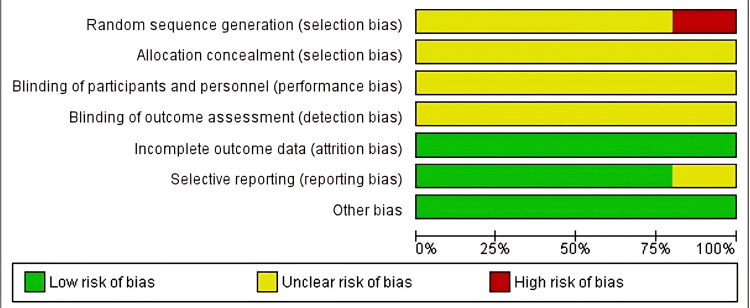
Risk of bias graph of the included randomised controlled trials (RCTs). References [24-28,31,38].

**Figure 3:**
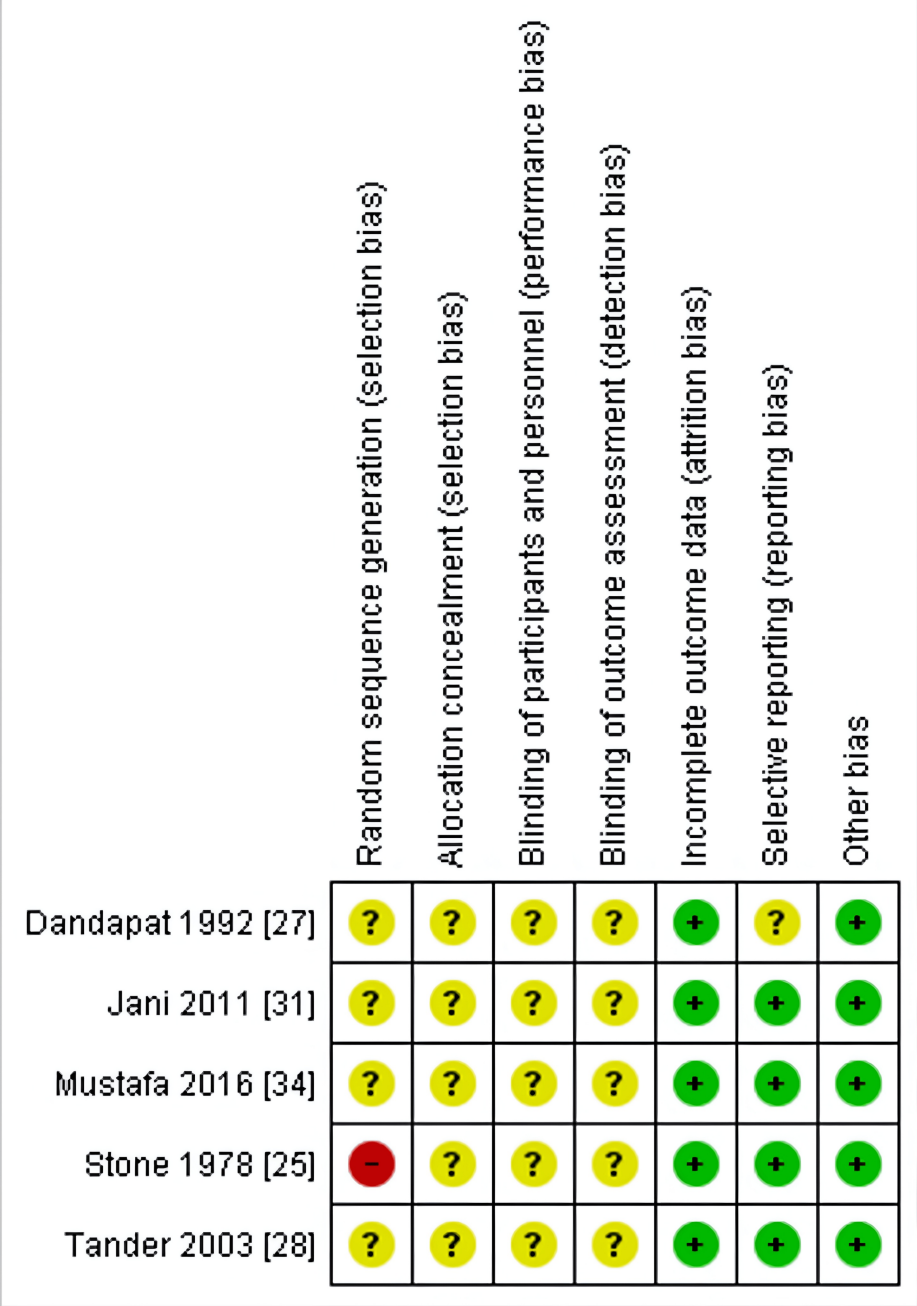
Risk of bias summary of included randomised controlled trials (RCTs). References [24-28,31,38].

**Figure 4:**
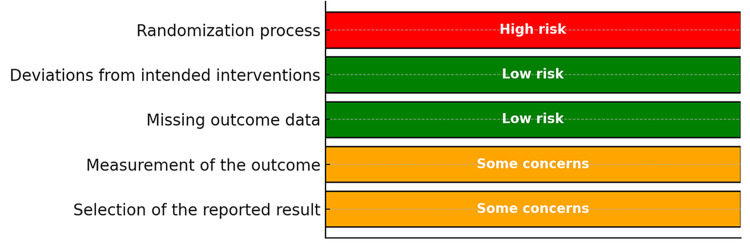
Cochrane Risk of Bias 2 tool. Tran et al. [24].

The Cochrane RoB2 tool [15] was utilised to evaluate the included RCTs (Figures 2, 3) [24-28,31,38]. The research conducted by Tran et al. [24] was evaluated using the RoB2 tool (Figure 4). An unclear or high risk of bias was found in these specific areas: random sequence generation and allocation concealment (selection bias), as well as for performance and detection bias. Attrition and reporting bias were unclear in one included study [27] but were of low risk in the remainder [24,25,28,31,38]. The study by Tran et al. [24] measured outcomes such as intra-abdominal abscess formation and LOS objectively; however, this study was judged to have some concerns. The assessment of wound infection/SSI was subject to observer bias (unblinded outcome assessors). Finally, no preregistered protocol was identified for this study (selective reporting concerns) [24].

Primary Outcome: Intra-abdominal Collection/Abscess Formation

**Figure 5:**
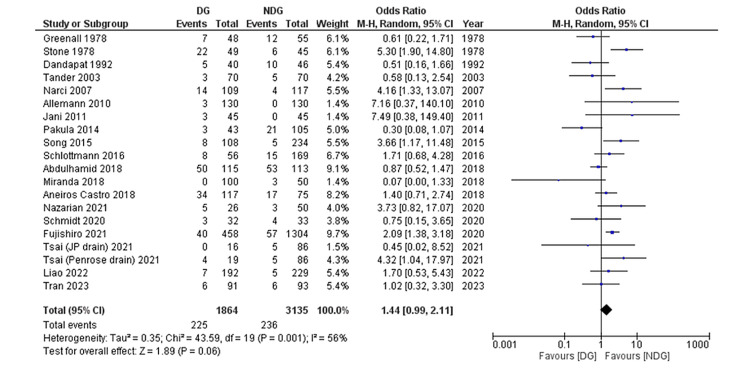
Forest plot of abdominal collection. References [22-37,39-41]. DG: drain group; NDG: no-drain group; M-H: Mantel–Haenszel.

Twenty studies involving 4,913 patients [22-37,39-41] reported post-operative intra-abdominal collection/abscess formation (Figure 5). A pooled analysis of these 4,913 patients showed no significant difference between the drain group (12.1%) and the no-drain group (7.5%) (OR = 1.44, 95% CI (0.99, 2.11), P = 0.06). The Cochran Q test indicated moderate heterogeneity between studies (I^2^ = 56%, P = 0.001).

**Figure 6:**
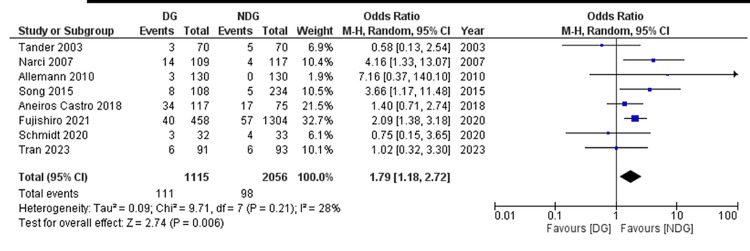
Forest plot of the subgroup for paediatrics-only studies, abdominal collection. Forest plot for the studies [24,28-30,33,36,39,40]. DG: drain group; NDG: no-drain group; M-H: Mantel–Haenszel.

Sub-group analysis for the paediatric population [24,28-30,33,36,39,40] showed a significantly higher rate of abdominal collections developing post-operatively in the drain group (9.9%) compared with the no-drain group (4.7%) (Figure 6) (OR = 1.79, 95% CI (1.18, 2.72), P = 0.006). The Cochran Q test revealed a low level of between-study heterogeneity (I^2^ = 28%, P = 0.21).

Secondary Outcomes: Surgical Site Infections (SSIs)

**Figure 7:**
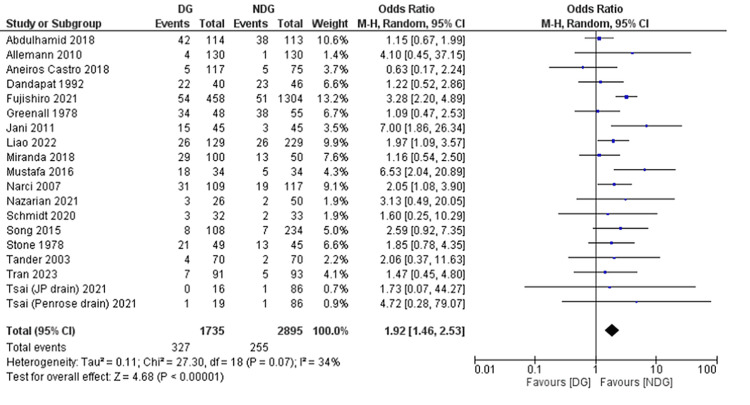
Forest plot of wound infection. References [22-31,33,35-41]. DG: drain group; NDG: no-drain group; M-H: Mantel–Haenszel.

This outcome was reported in 19 studies [22-31,33,35-41]. And it was significantly higher in the drain group (18.8%) compared with patients in the no-drain group (8.8%) (OR = 1.92, 95% CI (1.46, 2.53), P = 0.0001). The Cochran Q test revealed a low level of between-study heterogeneity (I^2^ = 34%, P = 0.07) (Figure 7).

Paralytic Ileus

**Figure 8:**
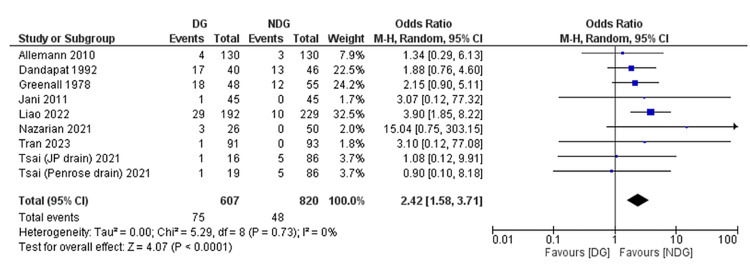
Forest plot of paralytic ileus. References [22-24,26,27,30,31,41]. DG: drain group; NDG: no-drain group; M-H: Mantel–Haenszel.

Paralytic ileus as an outcome was reported in nine included studies [22-24,26,27,30,31,41], and was 12.3% and 5.8% in the drain versus no-drain group, respectively (Figure 8). This was significantly greater in the drain group (OR = 2.42, 95% CI (1.58, 3.71), P = 0.0001). Between-study heterogeneity was low (I^2^ = 0%, P = 0.73).

Intestinal Obstruction

**Figure 9:**
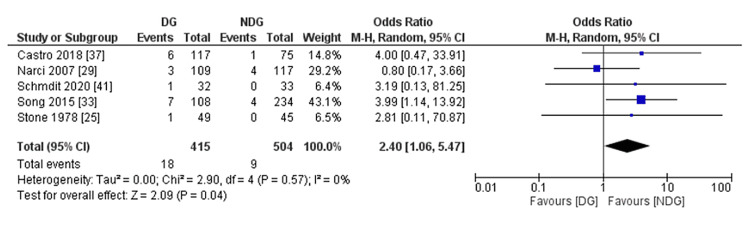
Forest plot of bowel obstruction. References [25,29,33,36,39]. DG: drain group; NDG: no-drain group; M-H: Mantel–Haenszel.

Intestinal obstruction was reported in five studies [25,29,33,36,39], with the rate being statistically higher in the drain group compared with the no-drain group (4.3% vs. 1.8%, OR = 2.40, 95% CI (1.06, 5.47), P = 0.04). Cochran Q test revealed a low level of between-study heterogeneity (I^2^ = 0%, P = 0.57) (Figure 9).

Length of Hospital Stay

**Figure 10:**
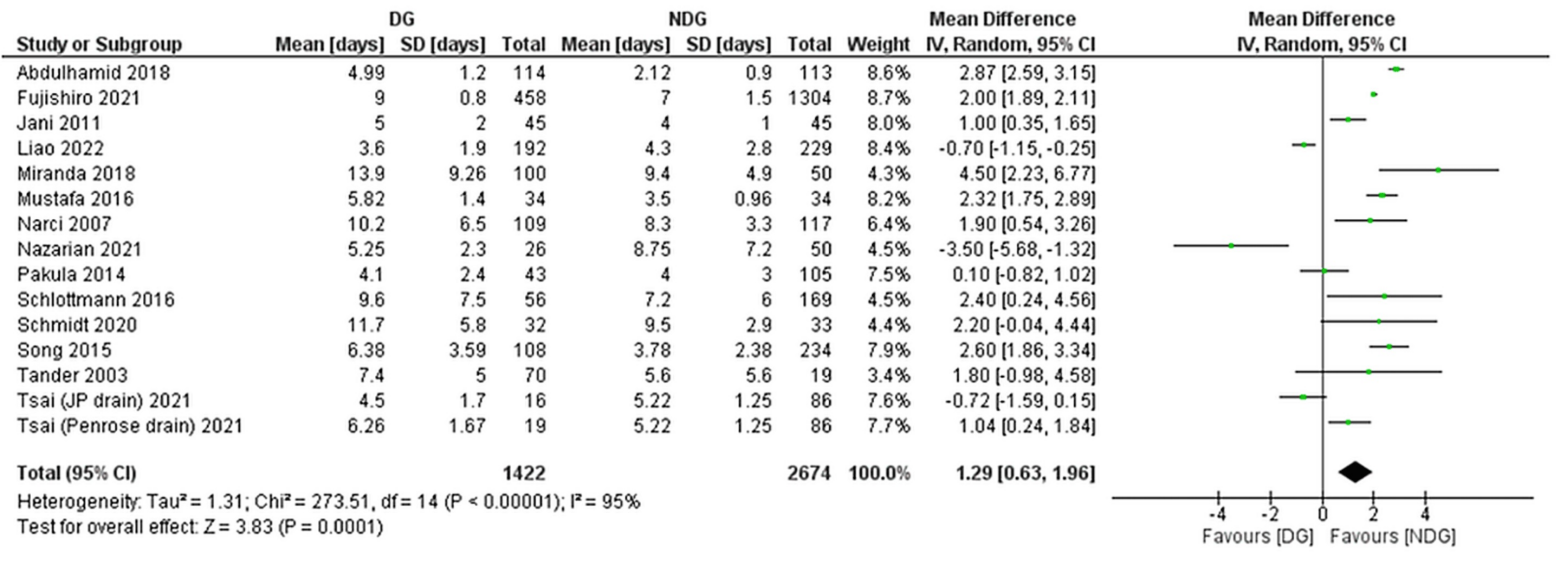
Forest plot of length of hospital stay. References [22,23,28,29,31-35,37-41]. DG: drain group; NDG: no-drain group; M-H: Mantel–Haenszel.

The LOS was reported as an outcome in 15 studies [22,23,28,29,31-35,37-41], involving 4,069 patients (Figure 10). A significantly shorter LOS was seen in the no-drain group compared with patients in the drain group (MD = 1.29, 95% CI (0.63, 1.96), P = 0.0001). Cochran Q test revealed a substantial level of between-study heterogeneity (I² = 95%, P < 0.00001), likely reflecting the inclusion of studies across several decades, different healthcare systems, and varying discharge criteria.

Mortality

**Figure 11:**
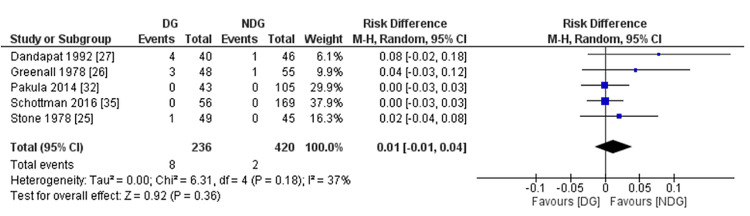
Forest plot of mortality rates. References [25-27,32,34]. DG: drain group; NDG: no-drain group; M-H: Mantel–Haenszel.

The mortality rate was reported in five of our included studies [25-27,32,34]. A pooled analysis of 656 patients did not reveal any significant difference in mortality between the two groups (3.4% in the drain group vs. 0.5% in the no-drain group, RD = 0.01, 95% CI (-0.01, 0.04), P = 0.36) (Figure 11). The between-study level of heterogeneity was low (I^2^ = 37%, P = 0.18).

Outcome Synthesis

Forest plots of outcomes are summarised in Figures 5-11.

Discussion

Acute appendicitis remains one of the most common indications for emergency abdominal surgery. Historically, prophylactic intra-abdominal drainage after appendicectomy for complicated appendicitis has been justified on the grounds of monitoring for haemorrhage or stump leakage and, critically, preventing post-operative intra-abdominal collections (IAC) [42]. However, a growing body of contemporary evidence challenges the efficacy of this practice and suggests that it may even be detrimental to patient outcomes [8,9,34,43,44].

The present study, comprising 21 studies and 4,963 patients, found that prophylactic drainage did not significantly reduce the incidence of IAC. This finding is consistent with multiple Cochrane reviews [8,43] and other meta-analyses [9,34,44], which have determined that the impact of drains on IAC is uncertain and provides no clear clinical benefit following open appendicectomy for complicated appendicitis. In fact, the most recent update raises concerns about a potential increase in 30-day mortality and increased LOS in patients with drains. However, the authors stress that this finding is based on low-certainty evidence [8,43]. This supports the conclusion that any presumed benefit of drains is likely negated by selection bias and the inherent morbidity associated with the drain itself [9,44].

Our paediatric subgroup analysis shows that placement of drains was associated with a higher rate of IAC. This is consistent with a recent paediatric meta-analysis [45] that reported an increased risk of IAC and longer LOS in the drain group, without a corresponding reduction in wound infection rates [45]. Together, these findings argue strongly against the routine use of drainage in children with perforated or gangrenous appendicitis and reinforce guideline recommendations cautioning against this practice [2,45].

Beyond the lack of benefit for IAC, our analysis identified a significant increase in harm, notably higher rates of SSIs in drained patients. This is consistent with two recent meta-analyses focused on laparoscopic management, which also found increased SSI rates and worse patient-centred outcomes, such as higher pain scores and prolonged LOS [10,34,44]. From a mechanical standpoint, this is plausible, as drains can act as a foreign body, serve as a conduit for bacterial contamination, and may fail to drain dependent collections effectively due to kinking or blockage, thereby adding device-related complications without any prophylactic effect [42].

The clinical disadvantages of drainage extend to post-operative recovery and the LOS. We observed higher rates of post-operative ileus and obstruction, as well as a consistently prolonged LOS with drain placement [9,10,23,34,44]. Based on this evidence, major clinical guidelines have undergone significant shifts. The World Society of Emergency Surgery (WSES) now recommends against the routine use of intra-abdominal drains after appendicectomy for complicated appendicitis in both adults (strong recommendation) and children (weak recommendation) [2].

This raises a practical question: What should a surgeon do when, in the midst of an operation, they have genuine concerns about bleeding or the integrity of the appendiceal stump? If a drain is deemed necessary in that situation, current evidence supports one clear approach, i.e., take it out as soon as safely possible (e.g., ≤48 hours) [46]. A recent retrospective study on laparoscopic appendicectomy for perforated appendicitis found that early removal of drains resulted in lower overall complication rates, shorter LOS, and reduced costs [46]. Whilst the study was non-randomised, it supports the approach of minimising drain dwell time. Prospective trials are needed to determine optimal timings for drain removal [8,43].

Our findings must be interpreted in light of several limitations. First, much of the contemporary literature is observational and therefore susceptible to confounding by indication; surgeons are inherently more likely to drain patients who have greater degrees of intra-abdominal contamination, biasing outcome comparisons [23,34]. Although many studies employed matching or multivariable adjustments to address this, the possibility of residual confounding still exists. Second, there is notable heterogeneity across studies in the definition of “complicated” appendicitis, criteria for diagnosing IAC and SSI, and details of drain management [9,10,23,34,44]. Third, the picture is further complicated by inconsistencies in surgical care. Factors such as the type of antibiotics used, the method of abdominal cavity irrigation, and whether a hospital has an Enhanced Recovery After Surgery (ERAS) program can all independently influence the results of a study. These limitations, reflected in the low GRADE (Grading of Recommendations, Assessment, Development, and Evaluation) ratings of existing evidence, emphasise the urgent need for an updated, adequately powered RCT (especially for laparoscopic surgery) that also captures patient-reported outcomes and cost-effectiveness [2,8,34,43,44].

Even with these limitations in mind, the evidence from randomised trials, high-quality observational studies, meta-analyses, and international guidelines converges on a consistent conclusion that routine prophylactic drainage after appendicectomy for complicated appendicitis offers no reduction in IAC and is associated with higher rates of SSI, ileus, obstruction, and prolonged LOS.

## Conclusions

Routine prophylactic intra-abdominal drainage after appendicectomy for complicated appendicitis offers no reduction in post-operative IAC and can be associated with morbidity. In paediatric cohorts, drains were linked to a significantly higher risk of IAC. Additionally, the use of drainage is associated with various adverse effects, such as a greater chance of SSI, increased likelihood of post-operative ileus and bowel obstruction, as well as extended LOS, with no clear advantages regarding mortality. Based on this evidence, routine prophylactic intra-abdominal drains should be avoided in both adult and paediatric patients after open or laparoscopic appendectomy for complicated appendicitis. When a drain is judged necessary in selected cases, current evidence supports removing it as early as safely possible, rather than leaving it in situ routinely. Future research should incorporate standardised definitions and protocols, including a definition for complicated appendicitis, antibiotic regimens, irrigation, indications for drainage, and timelines for removal. Additionally, patient-reported outcomes and cost-effectiveness should be considered. Until such data become available, a no-drain strategy remains the most evidence-based and patient-centred approach.
